# Endothelial Dysfunction through Oxidatively Generated Epigenetic Mark in Respiratory Viral Infections

**DOI:** 10.3390/cells10113067

**Published:** 2021-11-07

**Authors:** Spiros Vlahopoulos, Ke Wang, Yaoyao Xue, Xu Zheng, Istvan Boldogh, Lang Pan

**Affiliations:** 1 Horemeio Research Laboratory, First Department of Pediatrics, National and Kapodistrian, University of Athens, 11527 Athens, Greece; 2 Department of Microbiology and Immunology, University of Texas Medical Branch at Galveston, Galveston, TX 77555, USA; kewang@UTMB.EDU (K.W.); or xueyy459@nenu.edu.cn (Y.X.); zhengx807@nenu.edu.cn (X.Z.); sboldogh@utmb.edu (I.B.); 3 Key Laboratory of Molecular Epigenetics of Ministry of Education, School of Life Science, Northeast Normal University, Changchun 130024, China; 4 Department of Physiology, School of Basic Medicine, Xiangya Medical School, Central South University, Changsha 410078, China

**Keywords:** endothelial cells, oxidative stress, pulmonary edema, respiratory syncytial virus, influenza H1N1, SARS-Cov-2, respiratory distress syndrome, gene expression

## Abstract

The bronchial vascular endothelial network plays important roles in pulmonary pathology during respiratory viral infections, including respiratory syncytial virus (RSV), influenza A(H1N1) and importantly SARS-Cov-2. All of these infections can be severe and even lethal in patients with underlying risk factors.A major obstacle in disease prevention is the lack of appropriate efficacious vaccine(s) due to continuous changes in the encoding capacity of the viral genome, exuberant responsiveness of the host immune system and lack of effective antiviral drugs. Current management of these severe respiratory viral infections is limited to supportive clinical care. The primary cause of morbidity and mortality is respiratory failure, partially due to endothelial pulmonary complications, including edema. The latter is induced by the loss of alveolar epithelium integrity and by pathological changes in the endothelial vascular network that regulates blood flow, blood fluidity, exchange of fluids, electrolytes, various macromolecules and responses to signals triggered by oxygenation, and controls trafficking of leukocyte immune cells. This overview outlines the latest understanding of the implications of pulmonary vascular endothelium involvement in respiratory distress syndrome secondary to viral infections. In addition, the roles of infection-induced cytokines, growth factors, and epigenetic reprogramming in endothelial permeability, as well as emerging treatment options to decrease disease burden, are discussed.

## 1. Introduction

The endothelium is a semi-permeable barrier that separates any given tissue from blood or lymph. The endothelium nourishes every tissue and extends into all organs by forming a single-cell layer lining the inner surface of blood arterioles, capillaries, and post-capillary venules and lymphatic vessels. It covers thousands of square meters (up to 7000 m^2^) within an adult human. It forms the greatest surface where large numbers of physiological processes take place. The endothelial vascular network regulates exchange of fluid, electrolytes, various macromolecules (plasma proteins, hormones, inflammatory mediators), and controls trafficking of leukocyte immune cells. In addition, the endothelium maintains blood fluidity, regulates blood flow, and responds to signals triggered during oxygenation, hypoxia, and inflammation. Remarkably, endothelial cells can remain in a resting state over long periods of time, while keeping their ability to respond and regulate diverse processes when they are activated [1].

Dysfunction of the vascular endothelial barrier in the lungs can lead to acute respiratory distress syndrome (ARDS). ARDS is an extremely severe, frequently fatal condition characterized by fluid accumulation (edema) in the lungs due to a leaky endothelium under inflammatory conditions, induced by viral, bacterial or fungal infection. ARDS represents a great challenge for several patient populations. Presently, there is no efficient preventive measure or a means to reverse this fluid accumulation; essentially, supportive clinical care in terms of artificial ventilation is the standard treatment. In uninfected lungs, electrolytes and small polypeptides cross the intravascular space to the interstitial space via intercellular gaps between capillary endothelial cells and are returned by the lymphatics to the circulation. These electrolytes do not enter the alveoli in normal conditions due to the tightness of the epithelium in the alveoli [2].

## 2. Endothelial Cells

Endothelial cells (ECs) comprise the inner surface of blood vessels as a single-cell layer that has the function of a semi-permeable barrier between circulating blood and underlying tissue; ECs have a similar function in lymphatic vessels [3,4]. ECs regulate vascular function in terms of blood flow, blood fluidity, and vascular permeability, and are implicated in pathological manifestations after viral infections, control of immunity, inflammation, and leukocyte recruitment. Importantly ECs largely influence the spectrum of tissues that a virus can reach via circulation. ECs are effectors of the host response to viral infections; however, it must be noted that activation of host response to viruses occurs both in infected as well as uninfected cells, due to the diffusion of second messengers across intercellular gap junctions, and the secretion of paracrine mediators [5,6,7,8].

ECs regulate leukocyte traffic, vascular tone and permeability, and coagulation, and participate in control of allergic inflammation through the expression of soluble mediators and adhesion molecules in response to stimuli such as the cytokine interleukin-4 (IL-4) [9,10]. Expressed molecules also include: selectins E and P, intercellular adhesion molecules 1 and 2 (ICAM-1, ICAM-2), vascular adhesion molecule (VCAM), inflammatory cytokines interleukin-1 (IL-1) and -6 (IL-6), chemokines C-X-C motif chemokine ligand 8 (CXCL8, IL-8) and RANTES (Regulated upon Activation, Normal T Cell Expressed and Presumably Secreted), prostacyclin-2, endothelium-derived relaxing factor, nitric oxide, elements of local renin-angiotensin system, angiopoietins, tissue plasminogen activator, and endothelial plasminogen activator inhibitor [9,11,12,13,14]. Both in normal and inflamed tissues, ECs express CD antigens (cluster of designation), including CD31, CD34, CD309/Flk-1, CD202b/Tie2, CD144/VE-Cadherin, CD105/endoglin, CD146, acting as receptors or ligands [15,16].

The role of ECs in allergic responses that involve vascular damage and angioedema has been investigated in a clinical trial showing the role of ECs in chronic urticaria (registered with identification code NCT03443362), an inflammatory disorder that is driven by mast cells. EC dysfunction has a central role in this disorder, especially in the context of expression of adhesion molecules, increased vascular permeability, angiogenesis, increase in markers of coagulation and fibrinolysis, and importantly, increases in IL-4 and IL-6 [17].

### 2.1. The Pulmonary Endothelial Cell

Pulmonary ECs are an essential component of the gas exchange machinery of the lung alveolus, which can be divided into macrovascular and microvascular endothelial cells [18,19]. These cells are particularly interesting due to their potential for a number of important pathologic consequences upon infection, and during the process of recovery from infections or inflammation [20]. For instance, transdifferentiation of ECs or weakening of the endothelium, can occur. The latter can result from increased contractility and the loosening of intercellular tight junctions, which are both induced by inflammatory cytokines [21].

Abnormal activation of the host response to structures conserved among infectious agents and to known allergens activates the lung endothelium to elicit an angiogenic response associated with activation of T-helper cell type-2 (Th2)-driven inflammation [22]. Endothelial cells, as well as epithelial cells, actively participate in both innate and adaptive immune responses, which are crucially involved in the pathogenesis of allergic disorders; these responses are characterized by the expression and secretion of inflammatory mediators such as tumor necrosis factor alpha (TNFα) and by cytokines of the Th2 state such as interleukins-4 (IL-4) and-13 (IL13) [23].

In bronchi, endothelial and epithelial cells are also involved in pulmonary pathology [24]. The involvement of ECs in pulmonary pathology of viral infections is an active field of research. It has produced some controversial results yet promises to deliver important answers regarding the pathobiology of infectious disease. Specifically, endothelium has been shown to possess a key role in lung pathogenesis from viral infections such as influenza, respiratory syncytial virus (RSV), and recently SARS-CoV-2. A well-studied example is bronchiolitis associated with RSV infection [25].

### 2.2. Permeability of the Alveolar Endothelium during Respiratory Viral Infections

One potentially severe form of respiratory complication is manifested upon infection of bronchial endothelial cells. The endothelial loss of tight junctions (TJ) leading to edema in ARDS often causes alveolar epithelial damage due to the cells’ capacity to support RSV, influenza or CoV-2 replication. The loss of cell-to-cell contact in the endothelium is linked to death of alveolar epithelial cells, changes in extracellular matrix (ECM) protein components and their contact with endothelial and epithelial cells, and changes in the communication of both epithelium and endothelium with immune cells. Of note, TJs also modulate cellular polarity, activate a variety of intracellular signals, and control the transcellular transport across the endothelium and the epithelium by altering expression of transport proteins and ion channels. Lung inflammation is the key reason for TJ breakdown in the endothelium and epithelium [2,26,27].

Inflammation in the alveoli occurs prior to the development of endothelial breakdown of TJs and is associated with excessive changes in electrolytes and protein permeability [26]. In RSV, influenza, and CoV-2-infected experimental mouse models of acute innate inflammation and also in humans, this transition is characterized by excessive neutrophil influx, activation of macrophages, and exacerbated expression of type CC, CXC chemokines (IL-8, monocyte chemoattractant protein-1 (MCP-1 or CCL2), macrophage inflammatory protein-1 (MIP-1 or CCL3), cytokines (e.g., TNF-α), interleukins (IL-1β, IL-6), interferons (INFγ, IFNα and INFλ), and growth factors (e.g., granulocyte colony-stimulating factor, G-CSF). The reason is that most alveolar epithelial and endothelial cells express receptors for these soluble mediators and respond by amplifying the expression of these mediators [27]. For example, IL-1β increases endothelial and epithelial permeability via rat sarcoma virus homolog family, member A (RhoA)/integrins-mediated epithelial transforming growth factor beta (TGF-β) release. TGF-β signaling has been shown to induce post-translational modifications (e.g., phosphorylation) of adherent junction proteins and formation of actin stress fibers in endothelial cells in vitro [28]. IL-1β also enhances fluid transport across the human distal lung epithelium in vitro [29]. TNF-α strongly decreases the trans-endothelial electrical resistance across human pulmonary arterial endothelial cells; this effect is independent from myosin light chain phosphorylation catalyzed by either myosin light chain kinase or Rho serine-threonine kinase, but dependent on p38 mitogen-activated protein kinase activation [30]. The underlying mechanisms of capillary endothelial and epithelial barrier alterations point to apoptosis-dependent and apoptosis-independent mechanisms [31]. To this end, TNF-α also decreases expression of TJ proteins (ZO-1, claudin 2-4-5) as well as β-catenin in pulmonary arterial endothelium and also alveolar epithelium [32,33,34], which can be exacerbated by IFN-γ [35].

TNF-α also enhances human pulmonary microvascular endothelial permeability by altering the actin cytoskeleton via activation of PKC, increase of MAPK activity in a RhoA/ROCK-dependent manner, and inhibition of the Rho-dependent myosin-light-chain phosphatase [30,36,37]. TNF-α, IL-1β and IL-6 can upregulate trypsin expression in arterial endothelial cells, which may lead to the loss of zonula occludens-1 (ZO-1) and vascular hyperpermeability via protease-activated receptor-2 [38]. IL-4 and IL-13 decrease the expression of ZO-1 and occludin and lower the capacity of repair in the pulmonary epithelium, which also leads to compromise in arterial endothelial barrier function [39].

## 3. Respiratory Viruses and Their Effects on the Bronchial Endothelium

We next compare infections from RSV, influenza H1N1 and SARS-CoV-2 and describe their effects in the activation of inflammatory pathways that are implicated in bronchial pathology. The descriptions of these three viruses linked to dysfunction of the endothelial cells is not meant to be exhaustive, but instead provides examples of the severity of the threat for morbidity or loss of life that is associated with infections that compromise the function of the endothelial cells and can lead to dysregulation of the barrier function. We note that human rhinoviruses, parainfluenza viruses, metapneumovirus, respiratory adenovirus, bocaviruses, coronaviruses, middle-east respiratory syndrome virus (MERS), and severe acute respiratory syndrome virus (SARS) are also accountable for over three million deaths worldwide and are responsible for multiple outbreaks in recent times [40].

### 3.1. Respiratory Syncytial Virus

Infections by RSV are a formidable threat for certain groups of patients, especially newborns and elderly [25,41]. It is a single stranded, negative-sense enveloped RNA virus belonging to the *Orthopneumovirus* genus of thePneumoviridae family [42]. RSV replicates in the nasopharynx and then spreads within the epithelium of bronchi and bronchiole by cell-to-cell and producing clinical features including bronchiolitis and pneumonia [43,44,45]. RSV also productively infects non-epithelial cells; thus, it been isolated from alveolar endothelium, myocardial tissue, central nervous system, cerebrospinal fluid, endocrines and liver, during severe disease and sudden infant death [46,47,48,49]. There is no effective treatment or vaccine available for RSV; palivizumab (a humanized monoclonal antibody) is the only RSV immunoprophylaxis approved for use in specific high-risk pediatric populations [50]. The therapy is mostly supportive care combined with symptomatic treatments including modalities such as bronchodilators, epinephrine, corticosteroids, hypertonic saline, and/or supplemental oxygen [51,52].

Newborns cannot be expected to mount a sufficiently strong secretion of IFNs to respond against RSV, while the elderly do not respond well to vaccinations in general [53]. There are vaccine candidates under development, yet it remains a major challenge to immunize certain population categories [54]. It is imperative that therapies are developed to protect patients that either have not been vaccinated or cannot mount an optimal immune response to RSV. RSV infection causes respiratory symptoms that may encompass the lower respiratory tract, culminating in bronchiolitis, which in severe cases results in necrosis and the sloughing of epithelial cells into the airways, airway mucus, edema, and peribronchiolar inflammation, cumulatively resulting in airway obstruction [55,56,57]. Severe bronchiolitis is associated with the manifestation of asthma in later life [57]. Epithelial cells express cytokines IL-33, IL-25, and thymic stromal lymphopoietin (TSLP), as well as the innate immune cell-derived cytokine high mobility group box 1 (HMGB1), which activate group 2 innate lymphoid cells (ILC2). This signaling promotes the progression of T-helper type 2-mediated pulmonary disease, thus explaining the association of RSV with asthma in later life.

In vitro and in vivo experiments show that during RSV infection, epithelial cells infected with RSV express and secrete IL-1α, which activates vascular endothelial cells to express increased cell surface ICAM-1, and to a lesser extent, vascular adhesion molecule-1 (VCAM-1) and E-selectin [58]. RSV induces expression of MIP-1α in epithelial cells of the alveoli and bronchioles, as well as in adjoining capillary endothelium [59]. Adhesion experiments using polymorphonuclear leukocytes (PMN) verified an increased ICAM-1-dependent adhesion rate of PMN co-cultured with RSV-infected endothelial cells. Furthermore, the increased adhesiveness resulted in an enhanced transmigration rate of PMN [60]. ICAM-1 expression on RSV-infected endothelial cells may contribute to the enhanced accumulation of PMN into the bronchoalveolar space. The virus-induced ICAM-1 upregulation was dependent on the activity of protein kinase C, protein kinase A, phosphatidylinositol 3-kinase (PI3K), and p38 mitogen-activated protein kinase (MAPK) [60].

In lung alveoli, a gradient of CXCL8 is the most likely chemo-attractant for the neutrophils that migrate from the systemic circulation into the alveolar space [61]. Neutrophils function by releasing reactive oxygen species (ROS) and extracellular traps, undergoing degranulation and phagocytosis, and by recruiting other cell types to the site of infection such as alveolar macrophages, dendritic cells, and T-cells [62]. However, soluble endothelial cell adhesion molecules (sCAMs), such as sICAM-1, can be measured in the systemic circulation, indicating that the currently postulated neutrophil influx into the lungs should rather be regarded as a neutrophil efflux from the vasculature, involving substantial neutrophil-endothelial interactions. Endothelial cells become activated upon RSV infection, driving a ‘pro-adhesive state’ for circulating neutrophils with upregulation of endothelial ICAM-1. During RSV lower respiratory tract infections, different subsets of immature and mature neutrophils are present in the bloodstream, parallel with upregulation of integrins, lymphocyte-function associated (LFA)-1 and macrophage (Mac)-1 antigen, serving as ICAM-1 ligands [61].

RSV infection induces ROS generation, activates mitogen- and stress-activated kinases-1 (MSK1)-phospho-Ser-276 v-relreticuloendotheliosis viral oncogene homolog A (RelA) pathway required for cytokine expression [63]. Aero-allergens and respiratory viruses stimulate toll-like receptor (TLR) signaling, producing oxidative injury and inflammation [64]. Repetitive exacerbations produce complex mucosal adaptations, cell-state changes, and structural remodeling. These structural changes produce substantial morbidity, decrease lung capacity, and impair quality of life. Repetitive activation of innate signaling pathways produces a form of epigenetic ‘training’ in the cell nucleus, to induce adaptive epithelial responses [64].

### 3.2. Influenza Virus and SARS-CoV-2

Influenza viruses have a single negative-stranded segmented RNA genome; deadliest in history is H1N1, an *Alphainfluenzavirus* of the family *Orthomyxoviridae* [65,66]. SARS-CoV-2 in contrast, the causative agent of the COVID-19 pandemic, belongs to the positive-strand RNA viruses of the genus Beta coronavirus [67]. Both viruses constitute very significant health burdens worldwide, due to the lack of effective treatments, and are still under research for the generation of vaccines that will offer lasting protection against emerging variants [68,69]. In influenza virus infection, pulmonary endothelial cells play a central role in regulating both innate immune cell recruitment as well as innate cytokine and chemokine production [4]. In victims of the 2009 pandemic influenza A/H1N1 infection, tissues of bronchial mucosa, lung, myocardium, gastrocnemius, and liver that were investigated by light microscopy and transmission electron microscopy, viral particles were found in all samples, frequently located in endothelium, epithelium, and muscle cells [70]. Cultured ECs respond to infection and iron incubation with increased production of IL-6. Iron, the generation of intracellular hydroxyl radical and NF-κB activity are essential in cellular activation, suggesting that ROS generated in the Haber–Weiss reaction are essential in invoking an immunological response to infection by ECs [71].

In patients who died from SARS-CoV-2 or influenza (H1N1)-associated respiratory failure, the histologic pattern in the peripheral lung was diffuse alveolar damage with perivascular T-cell infiltration. The lungs from patients with COVID-19 also showed distinctive vascular features, consisting of severe endothelial injury associated with the presence of intracellular virus and disrupted cell membranes. Histologic analysis of pulmonary vessels in patients with COVID-19 showed widespread thrombosis with microangiopathy. Alveolar capillary micro-thrombi were 9 times as prevalent in patients with COVID-19 as in patients with influenza (*p* < 0.001). In lungs from patients with COVID-19, the amount of new vessel growth—predominantly through a mechanism of intussusceptive angiogenesis—was 2.7 times as high as that in the lungs from patients with influenza (*p* < 0.001) [72].

Although respiratory viruses initially infect the airway epithelium, it is a compromise in vascular integrity that causes alveolar damage [4]. Indeed, the compromise in vascular integrity distinguishes influenza H1N1 from SARS-CoV-2 infections, and this divergence in effects can be attributed to difference in the patterns of expression and secretion of inflammatory mediators. The main difference between influenza and SARS-CoV-2 infections is the ability of SARS-CoV-2 to elicit dysfunction of the blood vessels. This difference can be attributed to a divergent expression of signaling molecules that cause the pathology that involves blood vessels. A comparison between immunological factors produced during the influenza and SARS-CoV-2 infection suggests that although both infections raise levels of T-helper type I mediators, SARS-CoV-2 also distinctly increases T-helper type II (Th2) mediators (IL-4, IL-5, IL-10, IL-13), as well as the allergy mediator [73]. In contrast, H1N1 severe cases show high expression of surfactant protein D at the alveolar epithelium [73]. H1N1 infections have shown more efficient activation of reparative macrophages of the M2 subtype [74]. This might suggest a more efficient repair capacity for the H1N1-infected lung.

One hypothesis is that severe SARS-CoV-2-driven pneumonia causes respiratory failure via pulmonary microthrombi and endothelial dysfunction [75]. A considerable body of evidence suggests that SARS-CoV-2, unlike other related viruses, infects and replicates within ECs, which may explain a significant portion of the observed clinical pathology [76,77]. On the contrary, certain data that show an inability of SARS-CoV-2 to directly infect and lyse endothelial cells without angiotensin-converting enzyme-2 (ACE2) expression explain the lack of vascular hemorrhage in COVID-19 patients and indicate that the endothelium is not a primary target of SARS-CoV-2 infection [78]. Although the detection of SARS-CoV-2 has not been singularly linked to bronchiolitis, with the exception of necrotizing bronchiolitis [79], it has been proposed that co-infections of SARS-CoV2 with other viruses, most notably RSV, are associated with a severe course of bronchiolitis in patients [80]. Furthermore, SARS-CoV-2 can directly infect engineered human blood-vessel organoids in vitro. EC involvement was demonstrated across vascular beds of different organs in a series of patients with COVID-19 and SARS-CoV-2 can directly infect engineered human blood-vessel organoids in vitro [81].

The binding site of the SARS-CoV-2 viral spike protein on the surface of cells is the receptor “angiotensin converting enzyme 2 (ACE2)”, which functions to protect against hypertension, cardiovascular and lung diseases, and diabetes mellitus [82]. In an experimental setting, loss of ACE2 function in the mouse lung during endotoxin inhalation led to release of inflammatory chemokines such as C-X-C motif chemokine 5 (CXCL5), macrophage inflammatory protein-2 (MIP2), C-X-C motif chemokine 1 (KC), and pluripotent cytokine TNF-α from airway epithelia, increased neutrophil infiltration, and exaggerated lung inflammation and injury [83]. By immunohistochemistry, flow cytometry and RNA sequencing, the lung could show expression of ACE2, mainly in alveolar macrophages, and subsets of type II alveolar epithelial cells [84,85,86,87,88].

SARS-CoV-2 infection can result in diverse, multiorgan pathology, the most significant being in the lungs (diffuse alveolar damage in its different phases, micro-thrombi, bronchopneumonia, necrotizing bronchiolitis, viral pneumonia), heart (lymphocytic myocarditis), kidney (acute tubular injury), central nervous system (micro-thrombi, ischemic necrosis, acute hemorrhagic infarction, congestion, and vascular edema), lymph nodes (hemophagocytosis and histiocytosis), bone marrow, and vasculature (deep vein thrombosis) [79].

## 4. RSV, H1N1 and SARS-CoV-2 Infections and Oxidative Stress

**Figure 1:**
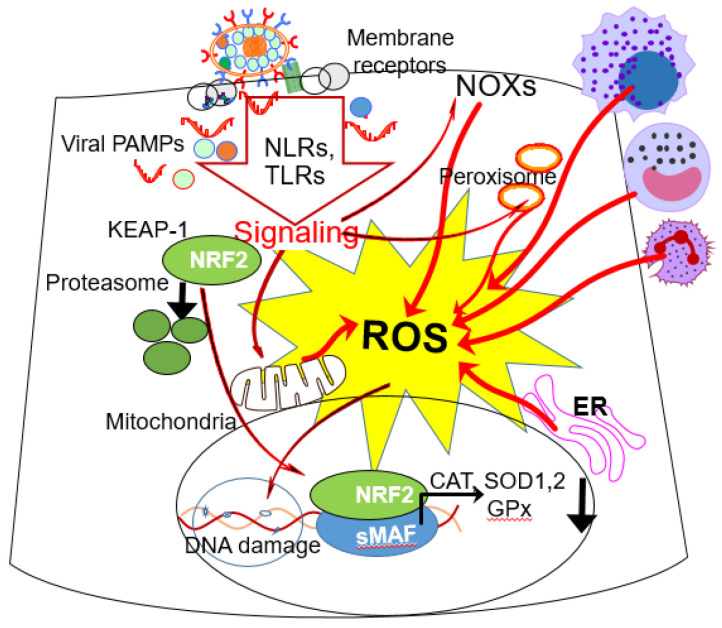
Cellular sources of ROS in respiratory viral infections. Activation of receptors liganded by RSV, influenza and SARS-CoV-2 envelope proteins and the recognition of viral PAMPs by intracellular sensors (NLRs, TLRs) trigger signaling pathways, leading to activation of oxidoreductases located in cell membranes, endoplasmic reticulum, peroxisomes, and mitochondria. NADPH oxidases (NOXs) are the primary enzyme complexes in nearly all cell types, particularly in granulocytes and macrophages, along with oxidoreductases in mitochondrial complex I and II, which partially oxidize oxygen molecules to generate superoxide anion (O_2_^•−^). O_2_^•−^ via Fenton and/or Haber-Weiss reactions are converted into hydroxyl radical (^•^OH). The highly reactive ^•^OH reacts with proteins, lipids and DNA. ROS themselves, but particularly peroxidation of polyunsaturated fatty acids, trigger nuclear translocation of nuclear factor erythroid 2-related factor 2 (NRF2), which heterodimerizes with small musculoaponeurotic fibrosarcoma (MAF) transcription factor and binds the cis-acting enhancer antioxidant response element (ARE), leading to the expression of antioxidant enzymes, including Cu/Zn-superoxide dismutase (SOD1), glutathione peroxidases (GPXs) and catalase (CAT). However, in RSV and SARS-CoV-2infected cells and lungs there is a progressive decrease in levels of NRF2 via increased protein ubiquitination and its degradation through a proteasomal pathway [93,99,100,101]. Although ROS generation in RSV, SARS-CoV-2 infected cells is similar, it seems that NRF2 primarily modifies influenza A entry and replication [102]. In addition to the above-described pathways, activated monocytes and polymorphonuclear cells, in particular, neutrophils, have been shown to produce ROS. Abbreviations: NLR, nucleotide-binding oligomerization domain-like receptors; TLR, TOLL-like receptors, PAMP, pathogen-associated molecular patterns; NOX, nicotinamide adenine dinucleotide phosphate (NADPH) oxidase; ER, endoplasmic reticulum.

Endothelial dysfunction is tightly correlated with oxidative stress, which represents unifying concepts for the underlying permeability changes and its pathophysiology marked by pulmonary morbidity and mortality [89,90]. Therefore, inflammatory cells are implicated in the induction of endothelial dysfunction, either directly from ROS generated by respiratory viral infections or indirectly by inflammatory mediators. The sub-molecular mechanisms of ROS generation are well established, and multiple sources are involved in generating oxidative stress during viral infections (Figure 1). These include mitochondria, peroxisomes, endoplasmic reticulum associated cytochrome oxidoreductases in nonimmune cells, and NADPH oxidase and xanthine oxidase in immune cells, as well as myeloperoxidase released from neutrophils [83,91,92]. In particular, RSV infection increases cellular ROS levels via NADPH oxidases and mitochondria, which are paralleled with the downregulation of superoxide dismutase, 1,3, catalase, and glutathione-S-transferase expression via degradation of the transcription factor NF-E2-related factor 2 [91,92,93]. Similarly, H1N1 infections increase ROS levels derived from multiple sources as determined by increases in oxidative metabolites such as 8-hydroxydeoxyguanosine (8-oxoGua), malondialdehyde, 2-isoprostane, 7-ketocholesterol, 7-beta-hydroxycholesterol, and carbonyl compounds as well as the activity of Nrf2, which controls the expression of enzymes that participate in the defense against oxidation [94]. In experimental animal models and cell culture infections with SARS-CoV-2, increases in ROS levels were observed through the activation of oxidoreductases, mitochondrial dysfunction and perturbation of cellular antioxidant defenses, similar to those of RSV or influenza infections [95,96,97]. Excess ROS generated by mitochondria is the primary cause of oxidative stress and is considered to initiate and exacerbate inflammation and chronic endothelial dysfunction [97]. Consequently, mitochondrial oxidative stress could prime endothelial cells to acquire a pro-thrombotic and pro-inflammatory phenotype, predisposing patients to thromboembolic and vasculitic events and to disseminated intravascular coagulopathy [97]. This implies a pivotal role played by oxygen centered free radicals in the pathogenetic mechanism of RSV-, H1N1-and SARS-CoV-2-induced diseases, in that its availability would tune the oxidant state and consequent damage [93,94,98].

## 5. Gene Expression Driving Pulmonary Pathologies via Dysregulation of Bronchial Endothelial Cells

Infected cells secrete soluble mediators such as inflammatory cytokines IL-1β, IL-6, and TNF, which in turn stimulate neighboring cells to express cascades of inflammatory mediators and adhesion molecules that enable exacerbation of inflammation, ROS generation and its associated pathological sequelae [82]. Endothelial cells, in response to viral infection and inflammation, express and secrete CXCL8, which activates neutrophil chemotactic movement and extravasation to exacerbate inflammation [103,104,105].

The inducible expression of cytokines and chemokines by inflammatory stimuli has been previously characterized. In particular, inflammatory stimuli induce a number of cell surface receptors and intracellular receptors, which in turn activate signaling cascades that culminate in the activation and nuclear translocation of transcription factors. These factors, bound to their DNA binding site, recruit transcriptional initiation and elongation components to activate expression of inflammatory genes in response to stimuli. The activated genes also encode several proteins that program the termination of inflammation to reestablish physiological cellular function. The return of cellular functions enables restoration of tissue homeostasis that is essential for the health of the organism [106].

Specifically, viral PAMPs through signaling cascades, and other inflammatory stimuli including ROS induce expression of cytokines and chemokines through activation of transcription factor binding on the promoter region of their genes [94,102,107]. In the activation of inflammatory gene expression, one of the best characterized transcription factors is nuclear factor kappa B (NFκB), which is activated after mediators of tissue stress, such as bacterial lipopolysaccharide, damage-associated molecules, ROS or inflammatory cytokines and cytokines that bind to their receptors at the cell surface [106,108]. These ligand-receptor interactions activate signaling cascades that culminate in the activation of gene expression by NFκB (and other transcription factors including STATs, AP1, and CREB), which, in turn, activates inflammatory genes such as chemokine IL-8 (CXCL8). IL-8 is secreted, generating concentration gradients at the host tissue that lead to the recruitment of neutrophils by chemotaxis [107,109]. Mechanistically, NFκB bound to its cis element, interacts with the transcriptional elongation complex, which consists of cyclin-dependent kinase 9 and bromodomain-containing protein 4 (BRD4). BRD4 facilitates phosphorylation of RNA polymerase II and regulates its enzymatic processivity and RNA splicing functions. Recent data also show that the association of RelA (NFκB’ catalytic subunit) with BRD4 induces its histone acetyl transferase activity acetylating histone H3 on Lys 122, which is a modification that leads to the destabilization of nucleosomes. Consequently, together this activity mediates cytokine production, neutrophilia, leukocytic infiltration, and clinical manifestations of disease [64].

In RSV, H1N1 or SARS-CoV-2 infection, clusters of NFκB target genes are expressed, driving the main part of pathological changes in tissues [64,110,111,112,113,114,115]. Respiratory viral infections result in inflammation and oxidative injury, as well as feedback-mediated enhancement of the expression of inflammatory genes. Although ROS are required for activation of NFκB and initiation of host antiviral responses, it has been shown that deregulation of the control of oxidant stress has a role in disease pathology, leading to the proposal of interventions that target the emergence of aberrantly increased ROS [116,117]. Therefore, the use of radical scavengers such as *N*-acetylcysteine and vitamin C, as well as inflammasome inhibitors, have been proposed as a method to inhibit the pathological effects of these viral infections [93,117,118,119].

Henceforth, it can be concluded that viral infection of airway epithelial cells, including type II cells, induces expression of inflammatory mediators, which are secreted and exposed at the cell surface. The mediators activate phenotypes of inflammation in all types of neighboring cells, regardless of the presence (or absence) of effective viral infections. This is especially true for pulmonary endothelial cells, which undergo extensive inflammatory changes in gene expression and phenotypes, which thereby intensifies the recruitment of inflammatory cells, like neutrophils, and damages host tissue [120]. Experimentally, activated vascular endothelial cells become a source of inflammatory cytokines and ROS, contributing to the development of coagulopathy, systemic sepsis, and cytokine storm.

## 6. Unifying Approach to Ameliorate Endothelial Dysfunction via DNA Occupancy of Transcription Factors

Because oxidative stress is the link to numerous pathologies caused by RSV, influenza, or SARS-CoV-2 infections, in theory, the use of antioxidants such as (vitamins E, C, SH group donors (e.g., *N*-acetyl cysteine), iron chelating agents (deferoxamine)- or activators of NRF2-driven gene expression, could be useful in decreasing expression of specific pro-inflammatory mediators and help recovery of experimental animals and patients from respiratory viral infections [91,93,99,100,117,119]. However, these attempts resulted in only partial successes to inhibit steps in the process of inducible expression of cytokines and chemokines and endothelial dysfunction [117,121,122].

Interaction of ROS with DNA produce modification to purine and/or pyrimidine bases and DNA strands (apurinic/apyrimidinic sites and DNA single- and double-strand breaks), which need to be repaired to maintain genome integrity. Of these, the most frequent is a purine oxidation product, 7,8-dihydro-8-oxodezoxyguanine (8-oxoGua), which is thought to be a pre-mutagenic lesion [123]. Under physiological conditions, it is removed via base-specific DNA repair enzymes primarily by 8-oxoguanine DNA glycosylase (OGG1) and to a smaller extent also by *Nei*-like glycosylases (e.g., NEIL1, NEIL-2, human homolog of *E. coli Nei*-like glycosylase 1and 2) from double and single stranded DNA, respectively [124]. The generated apurinic/apyrimidinic (AP) site is tailored by AP endonuclease1/redox effector factor-1 (APE1/Ref1) to form polymerase-ready 3’OH residues [124,125]. The generated DNA strand gaps are filled via the short and/or the long-patch repair sub-pathways [126].

**Figure 2:**
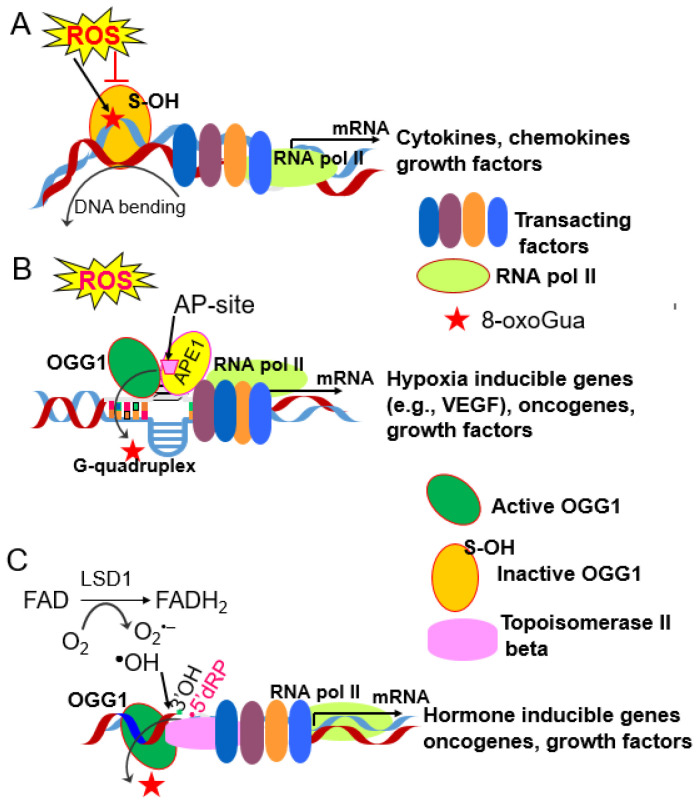
Models for OGG1-8-oxoGua-dependent gene expression. (**A**) Viral infection-induced ROS or those generated by cytokine exposure oxidatively modify guanine to 8-oxoGua and inactivate OGG1′ glycosylase activity by reversible oxidation at cysteine residues (cysteine-sulfenic acid). Oxidatively disabled OGG1 flips 8-oxoGua into its active-site pocket, interacts with the opposing cytosine and results in the conformational change of the DNA helix, which favors TFs DNA occupancy. (**B**) OGG1-8-oxoGua driven gene expression under hypoxic conditions. Guanines in gene promoters with G-quadruplexes are highly sensitive to ROS and are oxidized to 8-oxoGua under tissue hypoxia, caused by SARS-CoV-2, RSV, or H1N1 infections during pneumonia. OGG1 excises 8-oxoGua and generates an AP-site a substrate for APE1. APE1 binding leads to melting of the guanine duplex and stalls because of the non-canonical structure. Stalled APE1 increases transcription factor loading on the DNA via transient cooperative binding via conformational change of the helix. APE1, via its interacting domain, interacts with TFs (e.g., HIF1-α, STAT3, and CBP/p300) to modulate their redox state and promote both their binding to cis elements and gene expression. (**C**) OGG1-dependent transcription initiated by estrogens and its nuclear receptor. Estrogen (17β-estradiol; E2) binding to estrogen receptor alpha (ERα) results in demethylation of histone H3 lysine 9 (H3K9me2) via lysine-specific histone demethylase (LSD1; a flavin-dependent amine oxidase). Histone demethylation leads to a focal superoxide anion, hydroxyl radical generation and induces site-specific oxidation of guanine to 8-oxoGua. The latter is recognized and excised by OGG1 and via its AP-lyase activity cleave into the DNA strand generating the AP-site. The strand gap is recognized by topoisomerase II beta (topo IIb), which results in DNA structural changes in the chromatin allowing efficient assembly of transcriptional machinery and gene expression. Such scenarios are relevant to acute lung injury and SARS-CoV-2 infection capacity [138,139]. Similarly, LSD1-dependent DNA oxidation and OGG1 recruitment was needed for gene expression driven by TNFα, retinoic acid, and androgen exposure of cells [140,141,142]. Abbreviations: AP-site, apurinic/apyrimidinic site; APE-1, apurinic/apyrimidinic endonuclease 1; FAD, flavin adenine dinucleotide; FADH2, reduced flavin adenine dinucleotide; LSD1, flavin-dependent amine oxidase 1, 3-OH, 3-terminal hydroxyl; 5′-dRP, 5-terminal deoxyribose-phosphate.

Recent reports have documented that 8-oxoGua and AP-site, in gene regulatory elements of various inflammatory, hypoxia response genes and some proto-oncogenes, can modulate transcription [127,128,129,130,131,132,133]. For these reasons, 8-oxoGua(s) in a transcription start site (TSS) adjacent promoter sequences is considered as an epigenetic-like mark [129,133]. Binding of OGG1 to 8-oxoGua (with or without excision) in the gene regulatory region facilitates binding of transcription factors, including NFκB, as depicted in Figure 2A–C [132,134,135]. This 8-oxoGua enrichment in promoter regions is not unique to cytokine (e.g., TNFα)-exposed cells as promoters of hypoxia-inducible genes, including vascular endothelial growth factor and endonuclease III-like protein 1, which contains potential guanine-quadruplex forming sequences, which also acquire 8-oxoGua [128,129,136,137].

Inhibitors of OGG1 and 8-Oxogua Interactions in the DNA Helix Decreases Expression of Inflammatory Mediators and Inflammation

**Figure 3:**
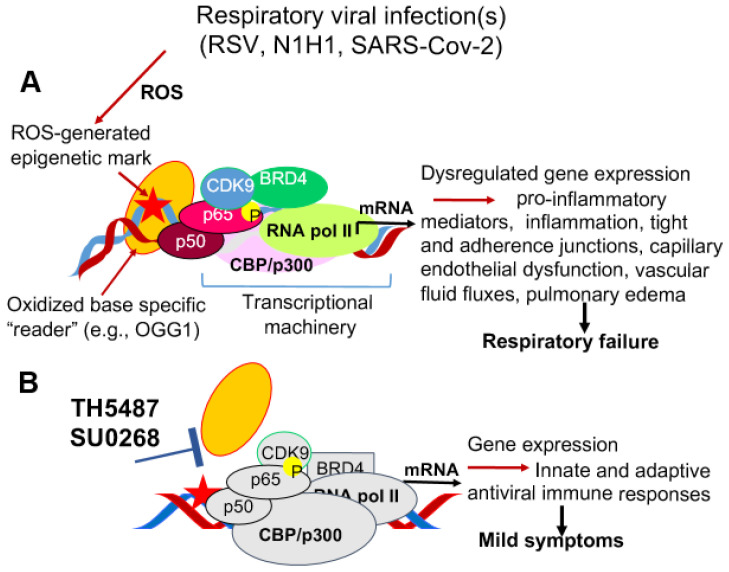
Proposed depiction of endothelial dysfunction by ROS in respiratory virus-infected airways and potential therapeutic intervention. (**A**) Oxidative modification to the heterocyclic DNA base guanine(s) in gene regulatory sequences is considered an epigenetic mark that is recognized by the “reader” OGG1, leading assembly of transcriptional complex and dysregulated gene expression. Consequences are pulmonary edema, congestion, respiratory failure in patients with risk factor(s). (**B**) Inhibition of OGG1′ interaction with epigenetic mark decreases extent of inflammation and manifestation of endothelial dysfunction. TH5487, and SUO268, OGG1 specific inhibitors; BRD4, bromodomain-containing protein 4; CDK9, cyclin-dependent kinase 9; p50-p65, nuclear factor kappa B; CBP/p300, RNA pol II, RNA polymerase II.

Studies described in Section 6, and references therein, show that OGG1, with or without 8-oxoGua excision, can regulate inflammatory responses (Figure 3A). Therefore, prevention of OGG1 interactions with genomic 8-oxoGua by inhibitors offer novel therapeutic opportunities (Figure 3). Pioneering studies by Dr. Lloyd’s laboratory have identified a family of hydrazide/acyl hydrazone inhibitor chemotypes (e.g., 3,4-dichloro-benzo[b]thiophene-2-carboxylic acid hydrazide), also known as O8, which inhibit OGG1-mediated catalysis at sub micromolar concentrations [143]. It allows OGG1 substrate interaction, but inhibits glycosylase activity, and thus stabilizes the OGG1-8-oxoGua complex. 

Another research group developed a chemically different compound, SU0268 that was shown to be selective for inhibiting OGG1 over other DNA base excision repair enzymes, and had dispensable toxicity in cultured human cell lines. The addition of SU0268 to cultured cells, inhibited OGG1 substrate binding and increased in intrahelical 8-oxoGua level [144,145,146]. SU0268 administration to experimental animals attenuate robust airway inflammation and increased survival rates after *Pseudomonas aeruginosa* infection. Remarkably, inhibition of OGG1 substrate binding increased type I IFN expression, which decreased bacterial load and disease progression [146].

Because OGG1-deficient mice are resistant to acute and systemic pulmonary inflammation, it was hypothesized that OGG1 inhibition may represent a strategy for the prevention and treatment. Therefore, a highly potent and selective small molecule inhibitor of OGG1, TH5487 [4-(4-Bromo-2-oxo-3H-benzimidazol-1-yl)-*N*-(4-iodophenyl) piperidine-1-carboxamide)] was developed [147]. The compound shows excellent membrane permeability and low levels of cytotoxicity in cultured cells and animals [147]. TH5487 precludes OGG1 binding to substrate-containing DNA in vitro and to genomic 8-oxoGua at nanomolar concentrations. Its administration to experimental animals challenged with bacterial lipopolysaccharides or TNFα decreases DNA occupancy of nuclear factor κB and suppress pro-inflammatory gene expression, airway endothelial permeability and inflammation [147,148].

Importantly, TH5487 is tolerated well when added repeatedly to experimental animals infected intrapulmonarily with RSV. TH5484 attenuated RSV-induced expression from inflammatory genes, accumulation of inflammatory cells and decreased endothelial permeability. Compared to untreated RSV-infected animals, histopathological analysis of Hematoxylin and Eosin (H&E) stained lung sections showed that inhibition of OGG1′ substrate binding decreased the density of perivascular inflammatory infiltrates (e.g., neutrophils, macrophages) and the number of inflammatory cells in alveoli, and their perivascular accumulation. Treatment with TH5487 of RSV-infectedor SARS-CoV-2infected human small airway epithelial cells (a relevant cell type to bronchiolitis) significantly decreased expression of over thirty pro-inflammatory genes including TNF, CCL20, IL6, CCL5 and CXCL10 at RNA and protein levels (GSE157630).

Overall, the small molecules TH5487 and SU0268 are first-in-class inhibitors of gene expression that depends on oxidative stress-generated epigenetic marks. They therefore may have clinical utility to lessen severe/chronic airway inflammation induced by respiratory viral infections. Of note is that the OGG1 inhibitor is used as a tool, and the possibility of using it in humans is speculative at this time.

## 7. Conclusions

Viral respiratory infections that cause endothelial dysfunction can have life-threatening consequences due to the central role of pulmonary endothelial cells in tissue homeostasis. Expression and secretion of soluble mediators and adhesion molecules generate inflammatory signaling cascades that shape the microenvironment of endothelial cells and compromises vascular integrity. Oxidative stress is increased in cells during inflammation and has a central function in the mechanism of activation of inflammatory gene expression. Although prevention of severe pathological sequelae is difficult, it is nevertheless feasible by interfering with the molecular mechanisms that induce inflammatory gene expression by viral infection. This type of molecular interference is already under clinical development. Finally, the recently developed small molecule OGG1 inhibitors, with nanomolar binding affinity, has provided a useful tool to test the role of oxidative stress-induced DNA base lesion(s) and OGG1 interactions, as rate limiting steps in violent cytokine storm and inflammation after viral and bacterial infection of the lungs.

## Data Availability

Cytokine and chemokinedata is deposited at National Center for Biotechnology Information, Gene Expression Omnibus (GEO) number: GSE157630.
